# Application of the surface engineered recombinant *Escherichia coli* to the industrial battery waste solution for lithium recovery

**DOI:** 10.1093/jimb/kuae012

**Published:** 2024-04-04

**Authors:** Jaehoon Jeong, Vidhya Selvamani, Murali kannan Maruthamuthu, Kulandaisamy Arulsamy, Soon Ho Hong

**Affiliations:** Department of Chemical Engineering, University of Ulsan, Namgu, Ulsan 44610, Republic of Korea; Department of Chemical Engineering, University of Ulsan, Namgu, Ulsan 44610, Republic of Korea; Department of Chemical Engineering, University of Ulsan, Namgu, Ulsan 44610, Republic of Korea; Department of Biotechnology, Indian Institute of Technology Madras, Chennai 600036, India; Department of Chemical Engineering, University of Ulsan, Namgu, Ulsan 44610, Republic of Korea

**Keywords:** Metal binding peptide, Lithium, Battery wastewater, *Escherichia coli*

## Abstract

*Escherichia coli* were engineered to selectively adsorb and recover lithium from the environment by employing a bacterial cell surface display strategy. Lithium binding peptide (LBP1) was integrated into the *Escherichia coli* membrane protein OmpC. The effect of environmental conditions on the adsorption of lithium by a recombinant strain was evaluated, and lithium particles on the cellular surface were analyzed by FE-SEM and XRD. To elevate the lithium adsorption, dimeric, trimeric, and tetrameric repeats of the LBP1 peptide were constructed and displayed on the surface of *E. coli*. The constructed recombinant *E. coli* displaying the LBP1 trimer was applied to real industrial lithium battery wastewater to recover lithium.

## Graphical Abstract

**Graphical Abstract:**
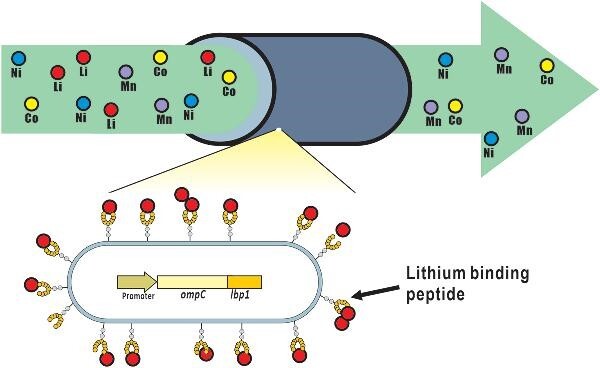

## Introduction

In recent years, a new discipline of mineral science called bio-hydrometallurgy or microbial mining (mining with microbes) has gained greater attention. Bio-hydrometallurgy deals with the application of biotechnology to the mining industry considering that mining with microorganisms is environment friendly (Diels et al., 1993; Haymore et al., 1992). Microorganisms utilized for metal recovery typically involve either a bio-leaching or biosorption process (Macaskie et al., 1987; Nguyen et al., 2013; Ravikumar et al., 2011). Bioleaching is the process of extraction or solubilization of minerals from ores by microorganisms, whereas biosorption deals with the cell surface adsorption of metals by microorganisms. For wastewater treatment, various functional microbes have been developed and intensively studied (Feng et al., 2022; Yin et al., 2022). Biosorption can be employed to recover valuable and toxic metals from industrial effluent, in spite of the non-specific adsorption. Metals can be selectively adsorbed by micro-organisms by employing the cell surface display of peptides, which have high specificity toward desired metal ions.

The cell surface display strategy has been applied for the extracellular display of proteins, peptides, and enzymes and has been widely employed in various fields including microbiology, biotechnology, and vaccinology (Bessette et al., 2004; Brown, 1992; Chen and Georgiou, 2002; Lee et al., 2003; Link and Tirrell, 2003). For the display of a desired specific peptide, the peptide needs to be integrated with the outer membrane proteins of a bacteria acting as an anchoring motif. Various membrane proteins, including outer membrane proteins, autotransporters, and S-layer proteins have been used as anchoring motifs for surface display (Agterberg et al. 1990; Cruz et al., 2000; Gurian-Sherman and Lindow, 1993; Han and Lee, 2015; Madhuranayaki, 2011; Xu and Lee, 1999; Yim et al., 2013). The display of proteins or peptides on the surface of a microorganism has become one of promising technologies for bioremediation (Diels et al., 1993).

Lithium is a soft silvery-white alkali metal that can be used in various industries, such as ceramics, glass, batteries, lubricants, polymers, and pharmaceutical industries. The importance of lithium getting higher these days based on the enormous demand for lithium-based batteries. The high charge and power-to-weight ratio of lithium make it an attractive component for powering electrical and electron devices. Lithium is required for the production of various types of batteries including lithium batteries, lithium-ion polymer batteries, lithium iron phosphate batteries, and nanowire batteries. Along with the increasing demand for lithium, the recovery and recycling of lithium from waste batteries have attracted more attention recently, considering the rising lithium price and the environmental toxicity of lithium battery waste (Ari, 2016). Generally, metals in low-concentration solutions can be recovered efficiently through the microorganism-mediated biological process, which makes the biological process an attractive method over physiochemical methods (Gadd, 1992).

A lithium-binding peptide (GPGAP)-displayed recombinant strain was constructed, and lithium adsorption studies were carried out (Selvamani et al., 2023). To understand the lithium binding mechanism and find a better lithium binding peptide, structural simulation was carried out. The peptide candidates were determined by Monte Carlo conformational analysis and quantum mechanical calculations of the binding energies of metal ions in solution.

In the present study, the selective recovery of lithium was carried out by the display of LBP1 (GPGNP), which has better lithium affinity than GPGAP peptide, on the surface of *Escherichia coli*. The lithium adsorption condition was optimized to maximize lithium recovery. The efficiency of the recombinant peptide (OmpC-LBP1) in lithium recovery was evaluated in Luria–Bertani culture medium (LB), and the adsorbed lithium was visualized by field emission scanning emission microscopy (FE-SEM) and transmission emission microscopy (TEM). Then the novel recombinant strain was tested in the artificial waste solution and the real industrial waste solution.

## Materials and Methods

### Bacterial Strains and Media

The *E. coli* strains used in this study are listed in Table 1 in the supplementary information. The strains were cultured and expressed in LB medium (10 g/L bacto-tryptone, 5 g/L bacto-yeast extract, and 5 g/L NaCl) with 100 mg/L ampicillin at 37°C with vigorous shaking at 250 rpm.

### Plasmid Construction

The recombinant peptide (OmpC-LBP1) was constructed by integrating *lbp1* at 993 bp of *ompC*. The recombinant construct *ompC*-*lbp1* was amplified by polymerase chain reaction (T100^TM^ Thermal Cycler, Bio-Rad Laboratories, Hercules, CA, USA) using nTaq-Tenuto polymerase (Enzynomics). The primers used in this study are listed in Table 2 in the supplementary information. The nucleotide sequence of the peptide LBP1 is GGCCCGGGCAACCCG and was designed based on codon usage. The construct *ompC*-*lbp1* was cloned into multiple cloning sites of the vector pET21a in between *NdeI* and *BamHI* (pETLBP1 × 1). A similar strategy was employed for the construction of dimeric (pETLBP1 × 2), trimeric (pETLBP1 × 3), and tetrameric (pETLBP1 × 4) repeats of peptide. The expression of the OmpC-LBP1 series was induced by the addition of isopropyl β-D-1-thiogalactopyranoside (IPTG) and regulated by the T7 promoter.

### Recombinant Protein Expression Analysis

The heterologous expression of the recombinant peptide OmpC-LBP1 was analyzed by sodium dodecyl sulfate-polyacrylamide gel electrophoresis (SDS-PAGE) (Laemmli, 1970). The recombinant *E. coli* displaying LBP1 was cultured overnight in LB medium at 37°C and diluted 100-fold in the same. The cultures were further incubated at 37°C until 0.5 optical density (OD) was recorded at 600 nm (OD_600_). The expression of OmpC-LBP1 was induced by the addition of IPTG. The concentration of IPTG (0–1.0 mM) was varied to optimize the expression condition. After the addition of IPTG, the cultures were further incubated at 30°C for 5 h. The recombinant strains were harvested by centrifuging cells at 13,000 rpm for 10 min. The protein was isolated by incubating cells with 700 µL 7 M urea buffer (7 M urea, 0.1 M NaH_2_PO_4_, 0.01 M Tris.cl pH8.0) at room temperature with shaking. The cell debris was removed by centrifuging the cells at 8,000 rpm. The isolated protein was analyzed by 12% SDS-PAGE and stained with Coomassie brilliant blue R-250 (Bio-Rad Laboratories, Hercules, CA, USA).

### Lithium Recovery Studies

After expressing the recombinant peptide OmpC-LBP1 series, the cells were harvested and washed twice with 0.85% (w/v) of NaCl. Recovery of lithium was carried out in LB medium at various temperatures (20°C, 25°C, and 30°C) and pH (1 and 7) with varying IPTG (0–1.0 mM) and lithium concentration (0.1–20 mM). Recovery of lithium was performed by incubating the recombinant strains in lithium-containing solutions for 30 min with shaking. Then the strains were washed twice with 0.85% (w/v) NaCl. The recovered lithium was extracted by further incubating the cells with 1 mM EDTA for 30 min on ice. The recovered lithium was analyzed using an AAS (AA-7000, Shimadzu, Kyoto, Japan).

### Modeling Studies

The possible sites of lithium binding by LBP1 were evaluated. A homologous model of OmpC-LBP1 was constructed using a modeler (Eswar et al., 2001). A search for potential structural templates was carried out using the AlphaFold2 tool.

### FE-SEM and TEM Analysis

After adsorption, the recombinant cells were washed with 0.85% NaCl (pH 5.8) to remove the unbound Li on the cell surface. Further, the cells were fixed and incubated in 2.5% glutaraldehyde at 4°C for 14 h. The fixed cells were washed with PBS and mounted on ultra-flat silicon wafers. The dried cells were visualized using FE-SEM and TEM.

### Lithium Recovery in NCM Solution

The *E. coli* (pETLBP1 × 3) strain was tested in an NCM (Nickel, Cobalt, and Manganese) artificial solution that contains each 1, 5, and 10 mM of NiCl_2_, CoCl_2_, and MnCl_2_ supplemented with the same concentrations of LiCl. The recombinant *E. coli* was incubated with each concentration of artificial waste water at 25 °C, 250 rpm for 30 min. After adsorption, cells were centrifuged at 10,000 rpm for 10 min and analyzed with ICP-OES.

The recombinant *E. coli* (pETLBP1 × 3) was cultivated in 10 mL LB media with a supplement of ampicillin. After overnight cultivation, 1% of cell broth was transferred into 100 mL LB media and incubated for 12 h at 30 °C, 250 rpm. The expression of the recombinant protein OmpC-LBP1 × 3 was induced with 0.5 mM IPTG at 37 °C, 250 rpm. Cells were collected by centrifuging at 5,000 rpm, washed twice with double-distilled water, and freeze-dried in FDU-2200 (Eyela, Tokyo, Japan). Freeze-dried cells were physically powdered and directly used in each amount (0.1–1 g) for adsorption of lithium in scrap waste water. Adsorption of lithium was studied at 25 °C for 3 h at 250 rpm and centrifuged at 10,000 rpm to collect the supernatant. Adsorbed lithium was analyzed with ICP-OES 5110 (Agilent, USA).

## Results

### Construction of Lithium Recovering System

**Fig. 1.**
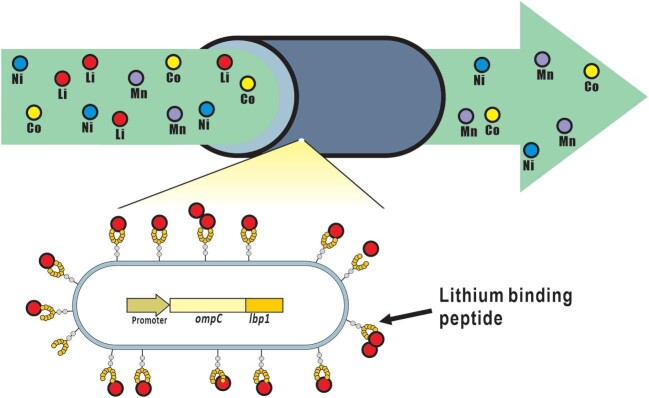
Schematic representation of this study. Recycle of lithium polluted waste water by recombinant *E. coli* displaying lithium binding peptide.

**Fig. 2.**
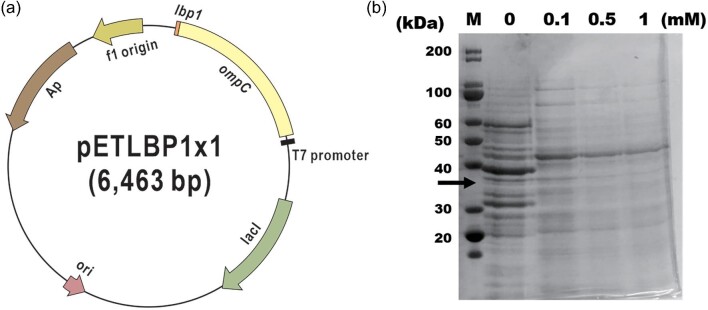
(a) Plasmid map of pETLBP1 containing lbp1 gene regulated by T7 promoter. (b) SDS-PAGE analysis of recombinant protein OmpC-LBP1×1 (M-Protein marker, IPTG concentration in mM).

A recombinant bacterial strain displaying short metal-binding peptides on the cellular surface was constructed and evaluated for the selective recovery of metals from the environment. In this study, lithium-binding peptide (LBP1) was integrated into the outer membrane-bound protein OmpC to construct the lithium-selective recovering recombinant *E. coli* (Fig. 1). The *lbp1* gene was attached to the truncated *E. coli ompC* in the eighth loop, and the dimeric, trimeric, and tetrameric repeats of the *lbp1* gene were also attached to enhance the lithium recovering efficiency (Fig. 2).

The expression of the recombinant peptide OmpC-LBP1 series was induced by the addition of IPTG to the recombinant strains. The effect of IPTG concentration on the expression was evaluated by culturing a recombinant strain at various concentrations of IPTG (0, 0.1, 0.5, and 1 mM). The expression levels were not changed dramatically by altering IPTG concentration; 0.1 mM IPTG showed the best expression level (Fig. 2b).

### Evaluation of Lithium Adsorption Conditions

**Fig. 3.**
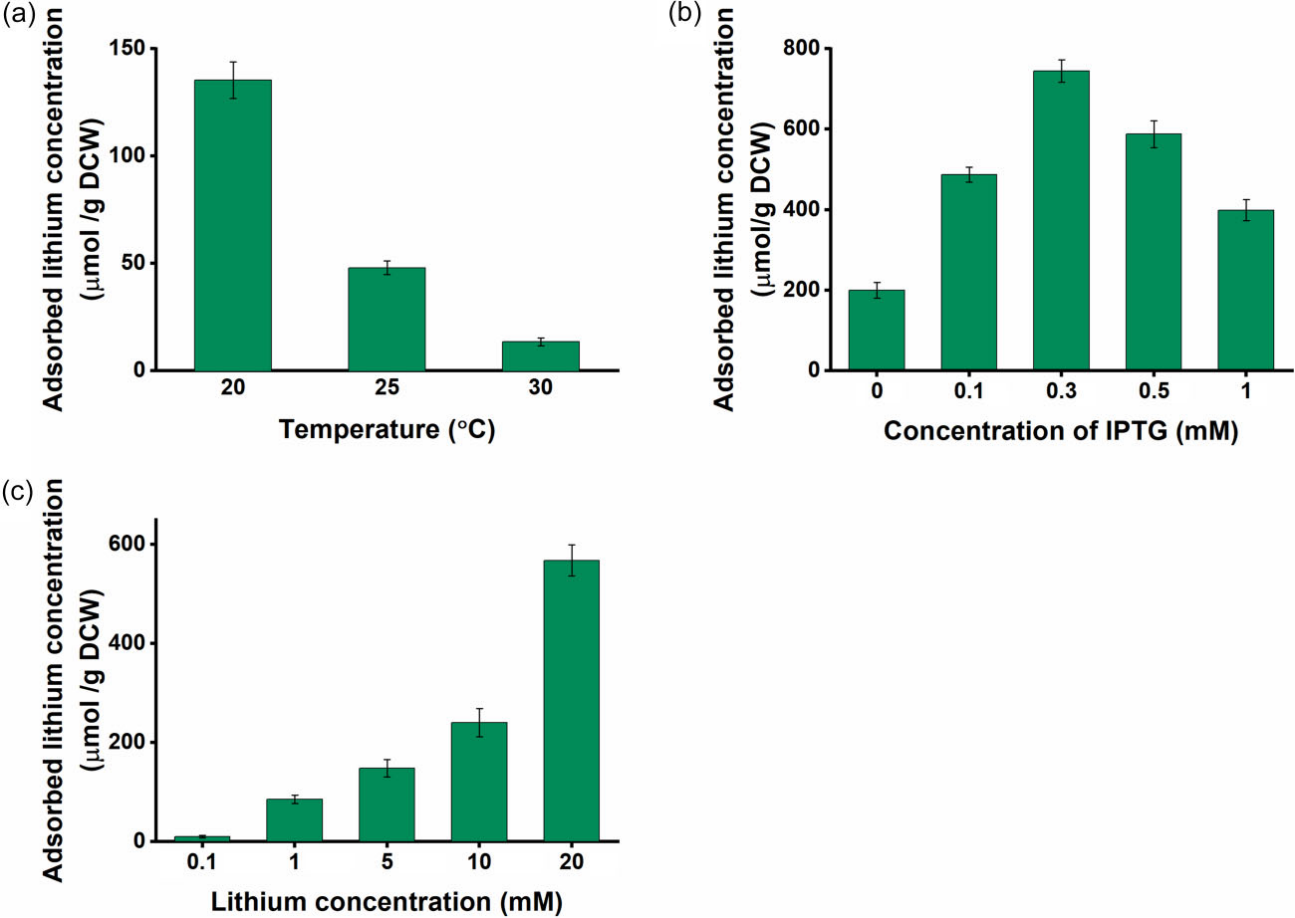
Lithium recovery studies with *E. coli* strains harboring pETLBP1 × 1 at various (a) temperature, (b) IPTG concentrations, and (c) lithium concentrations. All the experiments were independently performed in triplicates and standard deviation were determined.

The biosorption process is influenced by various factors including pH, temperature, initial metal ion concentration, and biomass or peptide concentration. These factors can either increase or decrease the amount of biosorption. To maximize the efficiency of the LBP1 displayed strain, various experimental conditions including culture temperature, IPTG concentration, and lithium concentration, need to be optimized. Culture temperature affects cell growth and protein folding. When the recombinant strain *E. coli* (pETLBP1 × 1) was cultured at different temperatures, the strain cultured at 20°C provided the highest lithium adsorption compared with those cultured at 25°C and 30°C (Fig. 3a).

The expression of the OmpC-LBP1 fusion protein was controlled by IPTG, and the IPTG concentration can affect the expression of the protein and lithium adsorption capacity. To evaluate the effect of IPTG concentration, a recombinant strain was cultured at various concentrations of IPTG (Fig. 3b). While IPTG concentration increased to 0.3 mM, lithium adsorption gradually increased. Then lithium adsorption decreased at a higher IPTG concentration. This data suggested that too high IPTG may cause metabolic burden and decrease cellular activity.

The effect of lithium concentration was also estimated by incubating the recombinant strain at various lithium concentrations (0.1–20 mM). Lithium adsorption was increased as lithium increased, and highest lithium adsorption was achieved at 20 mM lithium concentration (Fig. 3c). However, at higher lithium concentrations, lithium adsorption yield begins to decrease (data not shown). The effect of adsorption time was evaluated, and the result showed that maximum lithium adsorption was achieved within 15 min (data not shown). By finding the optimal temperature, IPTG concentration, and lithium concentration, the efficiency of the biosorption process can be maximized, leading to improved removal of lithium from contaminated wastewater.

### Physiochemical Characterization of Adsorbed Lithium

**Fig. 4.**
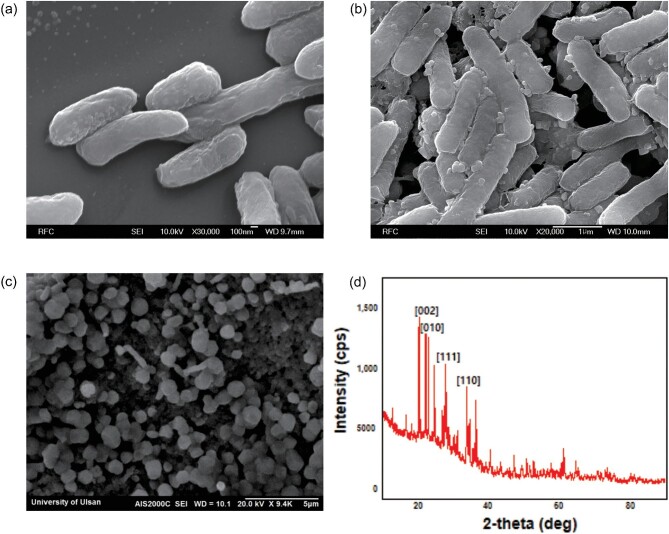
Cell surface analysis of recombinant strains containing OmpC-LBP1 after lithium adsorption. FE-SEM images (a) wild type *E. coli* and (b) recombinant *E. coli* after conducting lithium adsorption. (c) Calcinated recombinant *E. coli* displaying OmpC-LBP1 after conducting lithium adsorption. (d) X-ray diffraction (XRD) pattern of calcinated recombinant *E. coli*.

To visualize surface adsorbed lithium, the recombinant strains before and after lithium adsorption were observed by FE-SEM (Fig. 4a and b). The particles observed on the surface of cells were considered to be lithium, and the size of the lithium particles was around 25–100 nm (Fig. 4b). However, wild-type *E. coli* (BL21), which contained no lithium-binding sites, did not induce the formation of nanoparticles, although the cells were similarly treated with lithium solution (Fig. 4a). The nanoparticles in the SEM images of the recombinant *E. coli* may be inorganic nanoparticles of lithium salts. These results strongly support that lithium ions were adsorbed onto the surface of recombinant *E. coli* via the numerous binding sites, and lithium salt nanoparticles have formed near the cellular surface.

To obtain direct evidence of lithium nanoparticles, X-ray powder diffraction was performed after calcination of lithium adsorbed recombinant strain (Fig. 4c and d). After calcination, lithium nanoparticles with average size range of 0.6–0.9 μm were obtained (Fig. 4c). Various peaks of lithium oxide were observed, which indicate the polycrystalline nature of the product. Bragg's reflections for lithium oxide nanoparticles were observed at 2-theta values of 23.1^°^, 33.8^°^, and 36.4^°^ representing (002), (111), and (110) planes, respectively (Fig. 4d).

### Construction of LBP1 Multimer

**Fig. 5.**
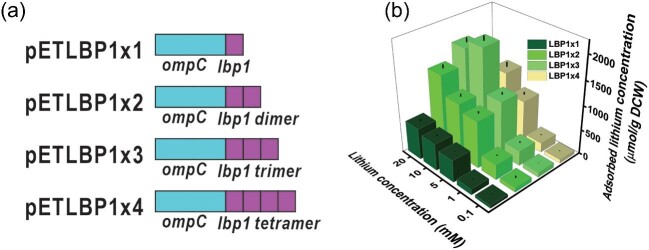
(a) Construction of recombinant plasmid for cell surface display of LBP2. (b) Adsorption study by OmpC-LBP1 monomer, dimer, trimer and tetramer displayed recombinant *E. coli* in various concentrations of lithium in LB medium.

In this study, a lithium-binding LBP1 peptide was attached to the membrane protein to selectively adsorb extracellular lithium. If two LBP1 were attached to a single anchoring motif, OmpC, theoretically, the lithium adsorption capacity of the cell surface would be doubled. To realize this idea and elevate the lithium adsorption capacity, dimer, trimer, and tetramer of LBP1 were attached to OmpC (Fig. 5a). To evaluate the effect of the LBP1 multimer on lithium adsorption, the recombinant *E. coli* strains harboring pETLBP1 series plasmids were incubated at varying concentrations of lithium (Fig. 5b). Among recombinant strains, the strain displaying the LBP1 trimer generally provided the highest lithium adsorption at most of the tested lithium concentrations. Recombinant *E. coli* (pETLBP1 × 3) adsorbed 1 976.2 µmol/g DCW of lithium at 10 mM lithium concentration. In case of the strains displaying LBP1 monomer and dimer, lithium adsorption increased as the lithium concentration increased. The strains displaying LBP1 trimer showed the highest lithium adsorption at 10 mM lithium concentration (Fig. 5b).

### Artificial NCM Waste Solution Analysis

**Fig. 6.**
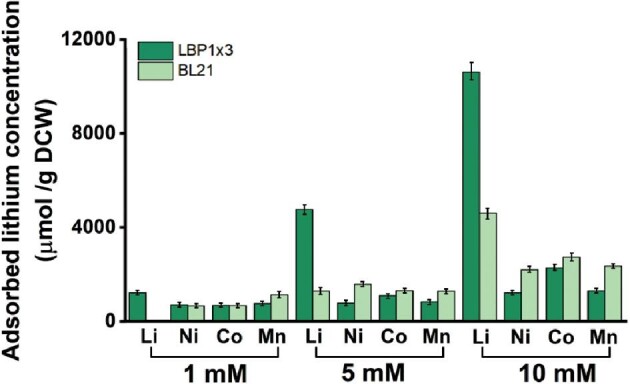
Lithium adsorption studies with (A) *E. coli* BL21(DE3) strain and *E. coli* (pETLBP1 × 3) in artificial NCM waste solutions containing various concentrations of Li, Ni, Co, and Mn ions.

The selectivity of the LBP1 displayed recombinant strain was evaluated by exposing the cells to an artificial NCM waste solution containing the same concentrations (1, 5, and 10 mM) of lithium, nickel, cobalt, and manganese ions. The recombinant strain showed higher selectivity toward lithium than other metal ions at every tested condition (Fig. 6). Recombinant *E. coli* (pETLBP1×3) adsorbed lithium at 10607.62 μmol/g DCW, 1206.858 μmol/g DCW, 2272.71 μmol/g DCW, and 1289.355 μmol/g DCW for nickel, cobalt, and manganese, which are 8.8, 4.7, and 8.2 fold higher than nickel, cobalt, and manganese present in artificial NCM waste solution, respectively (Fig. 6). These results indicated that LBP1 and its multimer showed high lithium selectively.

Based on the promising result obtained with an artificial NCM waste solution showing high lithium selectivity, the real-field application of the constructed system was evaluated. The recombinant strain was exposed to real NCM waste solution from the lithium battery recycling process (Fig. 6).

**Fig. 7.**
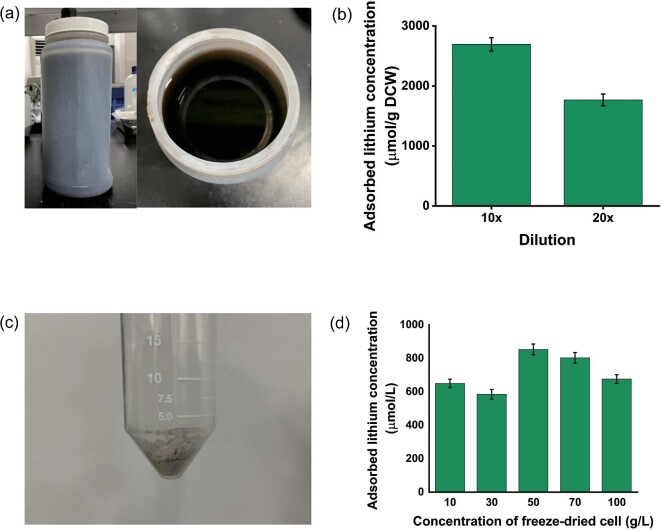
Lithium adsorption studies using real industrial battery waste solution. (a) Industrial battery waste solution. (b) Lithium adsorption studies carried out in 10- and 20-fold diluted battery waste solution. (c) Freeze-dried recombinant *E. coli* (pETLBP1 × 3) after LBP1 expression. (d) Lithium adsorption studies carried out in the real industrial battery waste solution using freeze-dried recombinant strain.

The concentration of lithium in the NCM waste solution was 7,641 ppm, or 1.1 M, which is enormously higher than that of previously used metal ion solutions (Fig. 7a). The effect of that high lithium concentration on cellular activity and the efficiency of lithium-binding peptides have not been fully studied yet. Therefore, the recombinant strain was tested in a 10- and 20-fold diluted NCM solution first to see the effects of high ion concentration and unknown composition on lithium adsorption by peptide (Fig. 7b). From the 20-fold diluted solution, 1,707 µmol/g DCW of lithium was adsorbed, which is about 15% of the initial lithium in solution while 2,731 µmol/g DCW of lithium was adsorbed from the 10-fold diluted solution with a recovery ratio of 12%.

These results showed that the recombinant strain can adsorb lithium in a real NCM solution. Considering the high concentration of lithium in the NCM solution, however, the current cell concentration is not high enough and we need to use more cells to absorb a meaningful amount of lithium. Therefore, the recombinant strain was freeze-dried after peptide expression to reduce cellular volume and increase volumetric cell concentration (Fig. 7c). From 10 to 100 g/L of freeze-dried cell was tested for lithium adsorption from real NCM solution. When 50 g/L of cell was used, 698.74 mM of lithium was adsorbed, which is about 37.9% of the initial lithium in solution (Fig. 7d). When higher concentrations of cells were used, less lithium was adsorbed, which may cause less effective mixing. The results reported here demonstrate that valuable metals can be recovered from real process NCM solution using designer peptides displayed on the surfaces of bacteria, inspite of the harsh conditions of the NCM solution toward bacteria.

## Discussion

The increase in electronic waste is directly proportional to the growth in the consumption of electronic goods and requires the development of novel recovery and reuse measures. Additionally, the increase in the use of lithium-ion batteries and poor current disposable systems demand the immediate development of lithium recovery systems. Therefore, the treatment of e-waste should be approached not only from the standpoint of cleaning but also mining.

We have shown that lithium sorbents can be prepared using five-amino acid peptides that exhibit a high binding specificity toward lithium and can extract it from solutions containing various metals under mild conditions without any harsh chemicals or energy input. The ability of these peptides to recover lithium can be bolstered by their display on the cell surfaces of recombinant bacteria.

**Fig. 8.**
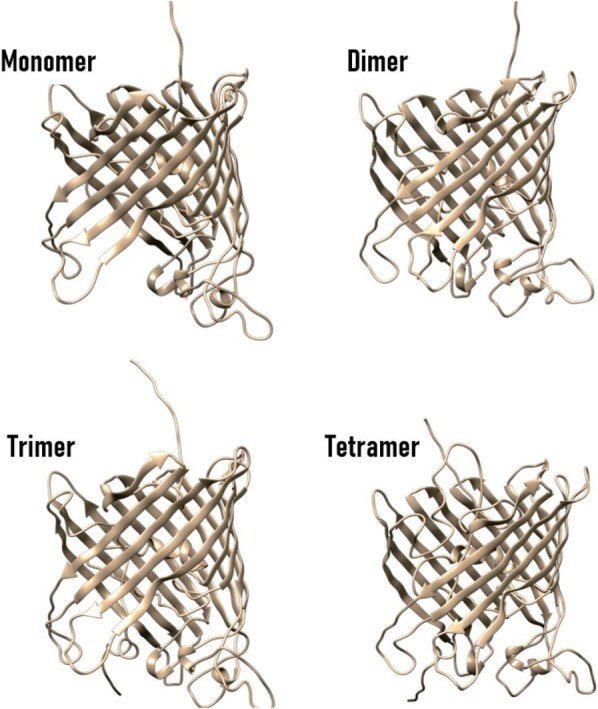
Three-dimensional structures of the four constructs, OmpC-LBP1 monomer, dimer, trimer and tetramer. Cyan and pink represent OmpC and LBP1, respectively.

The interaction with LBP1 and lithium ions was studied through structural prediction via AlphaFold2. Topological studies may explain the reduced lithium recovery in the tetrameric peptide construct. The predicted tree-dimensional structures of the OmpC-LBP1 monomer, dimer, trimer, and tetramer were created (Fig. 8). It was predicted that the monomeric, dimeric, and trimeric constructs had periplasmic LBP1 repeats, while in the tetrameric construct, two LBP1 repeats were in the transmembrane region and two in the extracellular region, potentially contributing to the diminished lithium recovery.

The real-world application of engineered bacteria raises concerns about potential ecological risks. These include the transfer of engineered genes to wild populations, unpredictable interactions with native organisms, persistence in the environment, impacts on non-target species, evolutionary pressures, and the potential for accidental releases. Effective monitoring, regulation, and ethical considerations are crucial to mitigate these risks and ensure responsible use of this technology.

Cell surface display of LBP1 peptide can reduce production costs and increase the adsorption capacity of recombinant strains. Furthermore, lithium from solution was recovered in the form of lithium nanoparticles; therefore, these whole-cell biosorbents can also function as templates for nanoparticle synthesis. The results presented in this paper should contribute to accelerating the development of economic lithium recovery processes.

## Supplementary Material

kuae012_Supplemental_File
