# Supplementary material for: Application of the surface engineered recombinant Escherichia coli to the industrial battery waste solution for lithium recovery
Source: J Ind Microbiol Biotechnol. 2024 Apr 4;51:kuae012. doi: 10.1093/jimb/kuae012 (PMC11037431; doi:10.1093/jimb/kuae012)
Supplement: kuae012_Supplemental_File [file kuae012_supplemental_file.docx]

**Supplementary Information**

**Table 1.** List of bacterial strains and plasmids used in this study

| Strain/Plasmid | Relevant genotype/ property | Source |
| --- | --- | --- |
| ***Escherichia coli* strains** | | |
| BL21 (DE3) | *F^–^ ompT gal dcm lon hsdS_B_*(*r_B_^-^ m_B_^-^*) *λ*(*DE3* [*lacI lacUV5-T7 gene 1 ind1 sam7 nin5*]) | Novagen |
| TOP10 | *F- mcrA Δ*(*mrr-hsdRMS-mcrBC*) *φ80lacZΔM15 ΔlacX74 nupG recA1 araD139 Δ*(*ara-leu*)*7697 galE15 galK16 rpsL*(*Str^R^*) *endA1 λ^-^* | Stratagene |
| **Plasmids** |  |  |
| pET-21a | Ap^R^ | NEB^a^ |
| pETLBP1x1 | pET-21a containing *ompC-lbp1* gene | This work |
| pETLBP1x2 | pET-21a containing *ompC***-***lbp1-lbp1* gene | This work |
| pETLBP1x3 | pET-21a containing *ompC***-***lbp1-lbp1-lbp1* gene | This work |
| pETLBP1x4 | pET-21a containing *ompC***-***lbp1-lbp1-lbp1-lbp1* gene | This work |

**^a^New England Biolabs, Beverly*,* MA, U.S.A.**

**Table 2.** Primers used in this work

| **Name** | **Sequence (5' to 3')** |
| --- | --- |
| LBP1_F | **CATATG**AGGAGAAATATAATGAAAGTTAAAGTACTGTCCCTCCTGGTC |
| LBP1x1_R | **GGATCC**GGGTTGCCCGGGCCCATGTTTTTGTTGAAGTAGTAGGTAGCACCAACATCAACATA |
| LBP1x2_R | **GGATCC**CGGGTTGCCCGGGCCCGCTTTCGCCGCCGCTTCCGCCGGGTTGCCCGGGCCCATGTTTTTGTTGAA |
| LBP1x3_OL_F | AAAACATGGGCCCGGGCAACCCGGCGGAAGCGGCGGCGAAAGCGGGCCCGGGCAACCCGGCGGAAGCGGCGGCGAAAGCG |
| LBP1x3_OL_R | CCGCTTCCGCCGGGTTGCCCGGGCCCGCTTTCGCCGCCGCTTCCGCCGGGTTGCCCGGGCCCGCTTTCGCCGCCGCTTCCGC |
| LBP1x3_R | **GGATCC**TTATTACGGGTTGCCCGGGCCCGCTTTC |
| LBP1x4_R | **GGATCC**GGATCCTTATTACGGGTTGCCCGGGCCCGCTTTCGCCGCCGCTTCCGCCGGGT |
